# Supplementary material for: The Evolution and Characterization of the RNA Interference Pathways in Lophotrochozoa
Source: Genome Biol Evol. 2024 May 7;16(5):evae098. doi: 10.1093/gbe/evae098 (PMC11114477; doi:10.1093/gbe/evae098)
Supplement: evae098_Supplementary_Data [file evae098_supplementary_data.zip › Supplementary_materials.zip!/selected_miRNAs/NoGe_selected_miRNAs/known_miRNA_families_pdf_files/NMRB01003818.1_Notospermus_geniculatus_strain_Ushimado_scaffold3818,_whole_genome_shotgun_sequence_44606.pdf]

Provisional ID	:	NMRB01003818.1_Notospermus_geniculatus_strain_Ushimado_scaffold3818,_whole_genome_shotgun_sequence_44606
Score total	:	46767.3
Score for star read(s)	:	3.9
Score for read counts	:	46758.7
Score for mfe	:	1.6
Score for randfold	:	0
Score for cons. seed	:	3
Total read count	:	91727
Mature read count	:	91624
Loop read count	:	5
Star read count	:	98

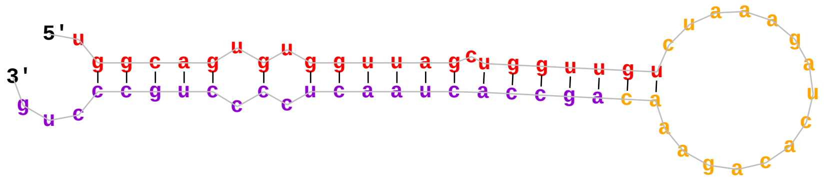

Genome coordinates: NMRB01003818.1 15604-15666

pre-miRNA sequence:
UGGCAGUGUGGUUAGCUGGUUGUUGGCAGUGUGGUUAGCUGGUUGUCUAAAGAUCACAGAAACAGCCACUAACUCCCCUGCCCUG

Mature sequence: UGGCAGUGUGGUUAGCUGGUUGU

Aligned mature sequence of the *N. geniculatus* pre-miRNA with the top 5 hits from the BLAST search against the pre-miRNA mirGeneDB set:

```
                              **********************
Nge_Mir-34-P12                uggcagugugguuagcugguugu-
Asu-Mir-34_5p                 uggcagugugguuagcugguugu-
Csc-Mir-34-P20_5p             uggcagugugguuagcugguugu-
Pmi-Mir-34_5p                 uggcagugugguuagcugguug--
Hme-Mir-34-P11_5p             uggcagugugguuagcugguuguu
Hme-Mir-34-P12_5p             uggcagugugguuagcugguuguu
```
